# The role of GABAergic receptors in acute, subacute, and withdrawal syndrome on pain and seizure thresholds in mice: A connection to mitochondrial function and oxidative stress in the brain

**DOI:** 10.1016/j.toxrep.2025.102158

**Published:** 2025-11-07

**Authors:** Roghayeh Jahani, Paria Pourbahram, Mohammad Seyedabadi, Fatemeh Nasiri, Hamidreza Mohammadi

**Affiliations:** a Student of Research Committee, Department of Toxicology and Pharmacology, Faculty of Pharmacy, Mazandaran University of Medical Sciences, Sari, Iran; b Department of Toxicology and Pharmacology, Faculty of Pharmacy, Mazandaran University of Medical Sciences, Sari, Iran; c Pharmaceutical Sciences Research Center, Institute of Herbal Medicines and Metabolic Disorders, Mazandaran University of Medical Sciences, Sari, Iran

**Keywords:** GABAergic receptor, Baclofen, Pain threshold, Seizure threshold, Oxidative stress, Mitochondria

## Abstract

One of the most serious neurological disorders is epilepsy. This study aimed to investigate the effects of baclofen, a GABA receptor agonist, on pain and seizure thresholds, as well as on oxidative damage in brain mitochondrial membranes of mice. Sixty male mice were divided into 10 groups. Control, baclofen (1, 5, and 10 mg/kg) with short-term exposure (1 day), long-term exposure (7 days), and withdrawal syndrome (eight days). The withdrawal syndrome was evaluated one day after the last dose of the drug. Hotplate and tail-flick tests were performed to assess pain threshold, and the rotarod was used to assess motor coordination. The seizure threshold and oxidative stress markers, including reactive oxygen species (ROS), malondialdehyde (MDA), protein carbonyl (PC), glutathione (GSH), and the MTT assay, were investigated. The results showed that baclofen (10 mg/kg) in short-term and all doses (1, 5, and 10 mg/kg) in long-term increased the seizure threshold. Evaluation of motor function and coordination in mice revealed decreased motor activity. The effect of baclofen on oxidative damage showed that, in long-term exposure, it improved mitochondrial ROS, malondialdehyde, and GSH levels. Protein carbonyl and MTT tests did not show a significant difference. A GABAB receptor agonist causes a dose- and time-dependent increase in the seizure threshold. Baclofen could reduce oxidative damage by decreasing ROS levels and malondialdehyde formation, and increasing GSH content.

## Highlights

• GABA_B_ receptor agonist increase the seizure threshold via dose and time-dependent manner.
• Baclofen in long-term administration, improved brain mitochondrial damage.
• Baclofen increase the pain threshold via muscle relaxant effect.
• Baclofen in the short term and long term increased the seizure threshold.
• Baclofen reduces oxidative damage by decreasing the oxidative damage in mitochondria

## Graphical Abstract

**Figure:**
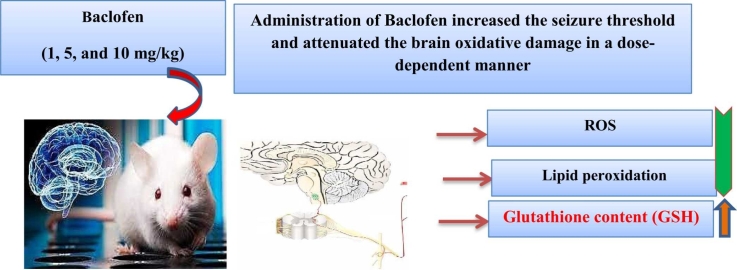
The effects of GABAergic Receptors in Acute, Subacute, and Withdrawal Syndrome on Pain and Seizure Thresholds in Mice.

## 1 Introduction

Seizure is a prevalent neurological condition, affecting about 7.6 out of every 1000 individuals over a lifetime. Seizures can affect brain function in specific areas and throughout the whole brain by using different mechanisms, leading to issues including impaired alertness, changes in cardiorespiratory function, and, in certain instances, even death [3], [6], [9], [21]. About 30–40 % of patients are resistant to all treatments and do not respond effectively to standard anticonvulsants [8]. The seizure threshold refers to the balance between excitatory and inhibitory signals in the brain. This threshold, which determines when a seizure may occur, varies from person to person [31], [37].

Pain is a complex experience shaped by factors such as tissue damage and environmental influences. It is one of the most common symptoms that prompts individuals to seek professional care in healthcare services [29]. Pain thresholds vary markedly among people when blunt pressure is applied to their muscles and joints. Low-pressure pain thresholds may signify a generalized state of pain sensitivity, variously attributed to altered sensory processing, dysregulated endocrine function, hyperinflammatory states, or psychological processes [32], [40].

Baclofen functions as a γ-aminobutyric acid (GABA) agonist, primarily binding to G-protein-coupled receptors [30]. It has central nervous system (CNS) depressant properties [42]. Baclofen is widely used in the clinical management of alcohol use disorder (AUD) patients, especially for those who do not respond to approved medications or who have severe liver disease. Baclofen may be recommended to assist patients with AUD in maintaining abstinence once it has been achieved or in attaining abstinence if they are currently still drinking. Patients should be warned about the increased risk of sedation when combining two sedative substances, such as alcohol and baclofen. Additional research is necessary to assess baclofen’s effectiveness and safety across various AUD patient populations (including women, adolescents, the elderly, and pregnant individuals), to determine the optimal treatment duration, and to understand better the risks associated with using alcohol and baclofen together [12].

GABA is the primary inhibitory neurotransmitter in the mammalian nervous system. It ideally needs to be in balance with glutamate to prevent nervous system dysfunction [44]. Overabundant glutamate and/or insufficient GABA can cause overexcitation in the CNS, leading to seizures [36]. One of the recognized mechanisms contributing to the onset of neuropathic pain is the disinhibition of dorsal horn neurons, mainly due to the disruption of the spinal GABAergic system [9]. Furthermore, reactive oxygen species (ROS) have been linked to the appearance of chronic pain conditions caused by nerve damage or inflammatory injury [47]. Studies showed that baclofen, a GABA derivative, can exert antispasmodic effects by activating GABA receptors to inhibit the transmission of synaptic reflexes [48]. An overabundance of Glutamate or a deficiency in GABA can contribute to epilepsy and pain. GABA receptors, which baclofen targets, are distributed throughout the central nervous system (CNS) [8]. This study aimed to investigate the effects of baclofen-induced GABA(B) receptor activation on pain threshold, motor coordination, seizure threshold, and oxidative damage in the brain mitochondria of mice.

## 2 Materials and methods

### 2.1 Chemicals and reagents

All chemical agents were purchased from Sigma-Aldrich, Chemie GmbH (Steinheim, Germany) (baclofen and pentylentetrazol, PTZ) and/or Merck Company (Germany). All reagents were of the highest purity and analytical grade.

### 2.2 Animals

Male BALB/c mice (n = 60; 20–25 g weight) were acquired from the Laboratory Animals Breeding Center, Mazandaran University of Medical Sciences, Sari, Iran. The animals were kept in (25 ± 1 °C) on a 12-h light:12-h dark cycle with free access to a regular diet. All experiments were conducted in accordance with the recommendations for animal experiments by the Ethics Committee of Mazanderan University of Medical Sciences (MAZUMS; Animal Ethical Committee, with research ethics certificate number: IR. MAZUMS.REC.1400.8929).

### 2.3 Animal treatment

In this study, the effect of different doses of baclofen (1, 5, and 10 mg/kg) in (1 and 7 days), as well as withdrawal syndrome (eight days), on the changes in pain threshold, seizure threshold, and oxidative damage were investigated in the brain mitochondria of the mice. The withdrawal syndrome was evaluated one day after the last dose of the drug. All of the 60 male mice were randomly divided into 10 groups (6 in each group) and treated as follows: control group (normal saline, IP), baclofen (1, 5 and 10 mg/ kg, IP; one day administration), baclofen (1, 5 and 10 mg/kg, IP; one week administration) and baclofen (1, 5 and 10 mg/kg, IP; withdrawal syndrome; one week treatments and then the treatments were stopped and tests were performed after the eight days.

The doses used in this study were determined based on a careful review of the literature [10], [38], [43]. Animals were anesthetized with ketamine/xylazine (10/1) and then sacrificed after behavioral tests. Oxidative stress biomarkers, including reactive oxygen species (ROS) formation, lipid peroxidation (LPO), protein carbonyl (PC) content, mitochondrial function, and glutathione (GSH) content, were measured in brain-isolated mitochondria.

### 2.4 Behavioral tests

#### 2.4.1 Hot-plate test

The hot-plate test assesses the nociceptive response to heat. Typically, temperatures of 52 or 55°C were used, with 48°C being less common. To keep the animal from jumping off the plate, a transparent plastic cylinder is positioned around it. As the surface temperature increased, the mouse may lick its paw or raise it. Elevated temperatures are not ideal due to the potential for burns. Licking the paw or lifting the claw is the measured parameter [20]. The cut-off time was established at 20 s to prevent tissue damage, and this duration was also regarded as the reaction time. This test was evaluated on the first, seventh, and eight days.

#### 2.4.2 Tail-flick test

For the tail flick study, mice were placed in a ventilated tube with their tails positioned over a wire coil at room temperature (23 ± 2°C). The flow of electric current subsequently increased the coil temperature, and the latency for the tail-withdrawal reflex was recorded. Radiant heat was administered to the tail at a distance of 5–8 cm from the tip using a tail flick device [22]. The heat stimulus was stopped after 10 s to prevent tissue damage (Cut-off time = 10 s). This test was evaluated on the first, seventh, and eight days.

#### 2.4.3 Balance and motor coordination

Rotarod was used to assess the quantitative motor performance, balance, and motor coordination in animals. The RPM of the device was adjusted so that the control group could remain stable on the rolling cylinders of the device for a maximum of 300 s [25].

#### 2.4.4 Determination of seizure threshold

Determining the PTZ-induced seizure threshold was investigated by inserting a 30-gauge butterfly needle into the tail vein of mice and infusing PTZ (1.0 %) at a steady rate of 0.5 ml/min into unrestrained animals. PTZ infusion was done 1 h after baclofen injection. The infusion was stopped when forelimb clonus was observed, followed by full clonus of the body. The lowest dose of PTZ (in mg/kg of mouse body weight) required to trigger a clonic seizure was noted as a measure of seizure threshold.

### 2.5 Mitochondrial tests

#### 2.5.1 Preparation of brain mitochondria

Mice were sacrificed, and their brains were removed immediately and washed with mannitol buffer. The brain tissues were homogenized (10 mM, pH 7.4), then centrifuged (2000 × g for 10 min; for the first time, the supernatant was carefully separated and centrifuged at 11000 g for 10 min at four ºC) to obtain the tissue mitochondria. For GSH, lipid peroxidation, and protein carbonyl tests, the brain mitochondria were suspended in Tris buffer or in a breathing buffer for measurement of ROS content [26].

#### 2.5.2 Measurement of total protein

The protein concentrations were determined using the Bradford method with Coomassie Blue [7]. The absorbance was measured at 595 nm by spectrophotometry (UV-1601 PC, Shimadzu, Japan)

#### 2.5.3 Mitochondrial ROS assay

2′,7′-dichlorofluorescein acetate (DCFH-DA) was used to measure ROS in isolated brain mitochondria. Briefly, DCFH-DA was added to the isolated mitochondria and incubated at 37°C for 15 min to allow the DCFH-DA probe to permeate membranes and for nonspecific esterases to cleave the diacetate groups. DCF formation was measured by a spectrofluorometer ( JASCO, FP6200, Japan) at excitation of 488 nm and emission of 525 nm wavelength at 37°C [27].

#### 2.5.4 Mitochondrial complex II activity assay

MTT assay indicated mitochondrial viability. Mitochondrial suspension with MTT was incubated at 37 °C for 30 min in the dark. Mitochondrial succinate dehydrogenase reduces this yellow indicator to the purple formazan, which is dissolved in dimethyl sulfoxide. The absorbance was measured at λ = 570 nm with an ELISA reader (Tecan, Rainbow Thermo, Austria) [46].

#### 2.5.5 Mitochondrial lipid peroxidation assay

Malondialdehyde (MDA) level, a marker of Lipid peroxidation, was measured using the thiobarbituric acid (TBA) reagent. We used mitochondrial suspension, phosphoric acid (85 %), and 25 μl TBA in a tube and heated for 30 min. Then, the samples were placed in an ice bath, and n-butanol was added to the contents of the tube. MDA involves the creation of TBA and red MDA-TBA2 by measuring absorbance at 532 nm using an ELISA reader (Tecan, Rainbow Thermo, Austria) [17].

#### 2.5.6 Mitochondrial protein carbonyl assay

To assess **protein carbonyl**, 200 μl of the isolated mitochondria suspension was combined with 0.5 ml of 20 % (w/v) trichloroacetic acid (TCA) in a microtube and then stored at 4°C for 15 min. After centrifugation, the sediment was combined with 0.5 ml of 0.2 % 2,4-dinitrophenylhydrazine (DNPH) and left at room temperature for 1 h. The samples were then rinsed with 1 ml of ethanol-ethyl acetate and centrifuged for 10 min, and the process was repeated three times. The precipitates were dissolved in 200 μl of guanidine HCl. Finally, absorbance was measured at 365 nm wavelength by an ELISA reader (Tecan, Rainbow Thermo, Austria) [26].

#### 2.5.7 Mitochondrial GSH assay

The mitochondria GSH content was calculated using 5,5′-dithiobis (2-nitrobenzoic acid) (DTNB) at 412 nm by an ELISA reader (Tecan, Rainbow Thermo, Austria). DTNB and TCA were added to the mitochondrial suspension, which appeared yellowish. The level of GSH was calculated via a standard curve of GSH and expressed as μM [23].

### 2.6 Statistical analysis

Statistical analyses were performed using GraphPad Prism version 9. One-way ANOVA followed by Tukey’s multiple comparisons test was performed. The data are presented as means ± SD (n = 6 mice per group). A two-way ANOVA was used to assess differences among groups. P < 0.05 was considered statistically significant.

For all Figures, significance notations are defined as: *** (p < 0.001), ** (p < 0.01), * (p < 0.05) versus the control group. Comparisons between other groups were analyzed via Tukey’s post hoc test.

## 3 Results

### 3.1 Behavioral tests

#### 3.1.1 Pain tolerance threshold

**Fig. 1:**
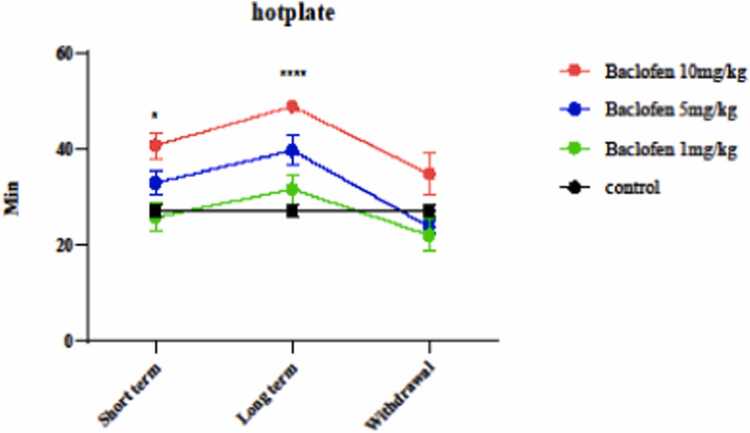
Effect of baclofen (1, 5, and 10 mg/kg) in short-term administration (1 day), long-term administration (7 days), and withdrawal syndrome (eight days) on pain tolerance threshold in the mice using Hotplate. Values are mean ± SD (n = 6 mice). * P < 0.05 compared with the control. ^****^ P < 0.0001 compared with the control.

**Fig. 2:**
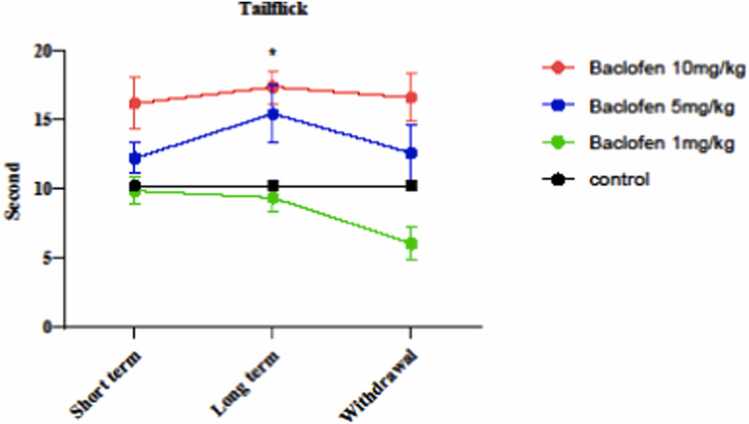
Effects of GABA receptor agonist (1, 5, and 10 mg/kg) in short-term administration (1 day), long-term administration (7 days), and withdrawal syndrome (eight days) on pain tolerance threshold in mice by Tail flick test. Values are mean ± SD (n = 6 mice). * P < 0.05 compared with the control.

Hot-plate and tail-flick tests were used to determine the effects of different doses (1, 5, and 10 mg/kg) of baclofen on the pain tolerance threshold in short-term (1 day), long-term (7 days), and withdrawal (8 days) exposures. The results are in Fig. 1, which shows that a 10 mg/kg dose of baclofen significantly increased the pain threshold on the first day (p < 0.0169) and the seventh day (p < 0.0001) of the injection period compared to the control group. Fig. 2 indicates pain threshold changes using the Tail Flick Test. 10 mg/kg on the seventh day of the injection period caused a significant increase (p < 0.0316) in the response to pain stimuli compared to the control group.

#### 3.1.2 Seizure threshold

**Fig. 3:**
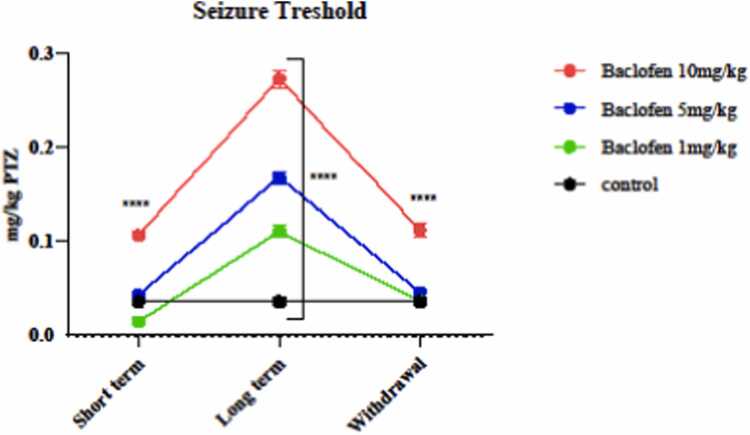
Effects of GABA receptor agonist (1, 5, and 10 mg/kg) in short-term administration (1 day), long-term administration (7 days), and withdrawal syndrome (eight days) on seizure threshold in the mice. Values are mean ± SD (n = 6 mice). ^****^ P < 0.0001 compared with the control.

To evaluate the seizure threshold, one hour after treating animals with different doses of baclofen (1, 5, and 10 mg/kg), PTZ was injected, and seizure threshold was evaluated on the first and seventh days of the injection, and withdrawal syndrome (eight days), and compared with the control group. Fig. 3 shows that 10 mg/kg of baclofen significantly (p < 0.0001) increased the seizure threshold on the first and seventh days of the injection and withdrawal phases of the syndrome in mice. 1 and 5 mg/kg on the seventh day of the injection caused a significant increase in seizure threshold compared to the control group (p < 0.0001).

#### 3.1.3 Activity and motor coordination

**Fig. 4:**
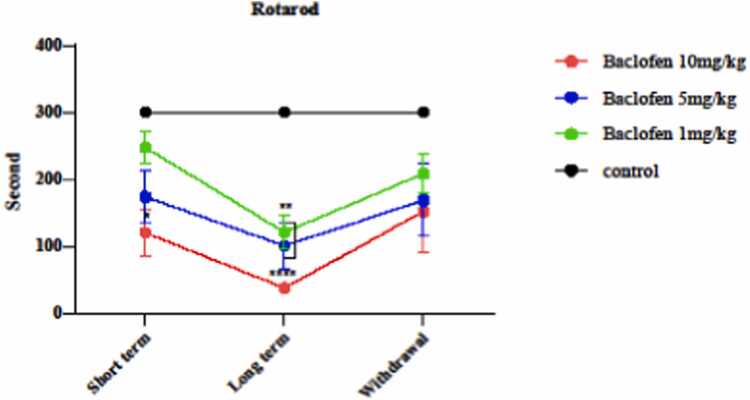
Effects of GABA receptors agonist (1, 5, and 10 mg/kg) in short-term administration (1 day), long-term administration (7 days), and withdrawal syndrome (eight days) on activity and motor coordination in mice by Rotarod. Values are mean ± SD (n = 6 mice). * P < 0.05 compared with the control. ^**^ P < 0.01 compared with the control.^****^ P < 0.0001 compared with the control.

Rotarod was used to investigate the effect of baclofen on the activity and motor coordination in mice. As shown in the Fig. 4, baclofen (10 mg/kg) on the first day (p < 0.0285) and on the seventh day of the injection at one (p < 0.0016), 5 (p < 0.0041), and 10 mg/kg (p < 0.0001) caused a significant decrease in motor strength in mice compared to the control group.

### 3.2 Oxidative stress markers

#### 3.2.1 Effect of baclofen on ROS level

**Fig. 5:**
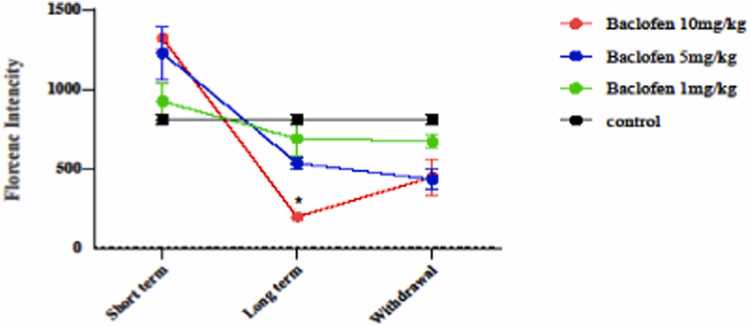
Effects of GABA receptor agonist (1, 5, and 10 mg/kg) in short-term administration (1 day), long-term administration (7 days), and withdrawal syndrome (eight days) on the level of reactive oxygen specious (ROS) in the mitochondria of the mice brain. Values are mean ± SD (n = 6 mice). * P < 0.05 compared with the control.

The level of ROS after exposure to different doses of baclofen in short-term, long-term, and withdrawal conditions is shown in Fig. 5. The findings showed that ROS level significantly reduced in 10 mg/kg on the long-term of the injection (p < 0.0321) in the mitochondria of the mouse brain compared to the control group.

#### 3.2.2 Effect of baclofen on lipid peroxidation level

**Fig. 6:**
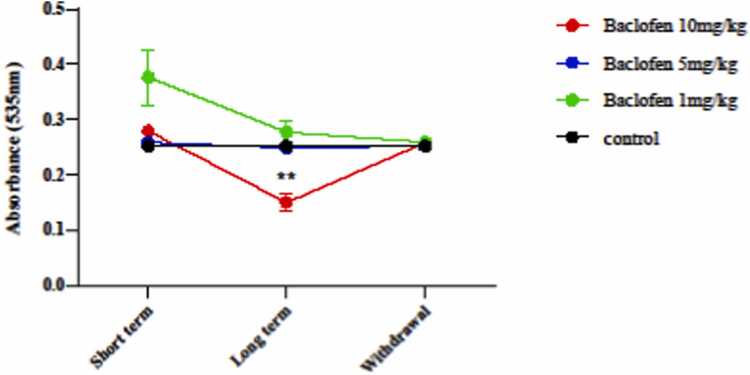
Effects of GABA receptor agonist (1, 5, and 10 mg/kg) in short-term administration (1 day), long-term administration (7 days), and withdrawal syndrome (eight days) on the level of malondialdehyde (MDA) as a lipid peroxidation marker in the mitochondria of the mice brain. Values are mean ± SD (n = 6 mice). ^**^ P < 0.01 compared with the control.

The level of MDA after exposure to different doses of baclofen in short-term, long-term exposure, and withdrawal syndrome is shown in Fig. 6. The results showed that the amount of MDA decreased by 10 mg/kg in the long-term injection group (p < 0.03) in the mouse brain mitochondria compared to the control group.

#### 3.2.3 Effect of baclofen on GSH level

**Fig. 7:**
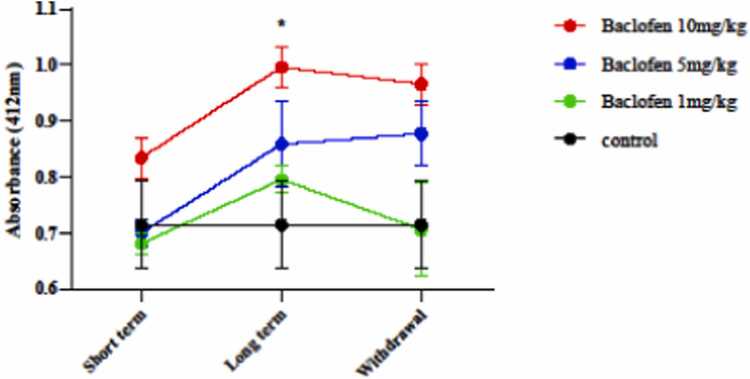
Effects of GABA receptor agonist (1, 5, and 10 mg/kg) in short-term administration (1 day), long-term administration (7 days), and withdrawal syndrome (eight days) on the level of Glutathione content (GSH) in the mitochondria of the mice brain. Values are mean ± SD (n = 6 mice). * P < 0.05 compared with the control.

The level of GSH after exposure to different doses of baclofen in short-term, long-term exposure, and withdrawal syndrome is shown in Fig. 7. The findings showed that GSH levels significantly increased at 10 mg/kg over the long term of the injection (p < 0.0213) in the mitochondria of mouse brains compared to the control group.

#### 3.2.4 Effect of baclofen on mitochondrial function (succinate dehydrogenase activity)

**Fig. 8:**
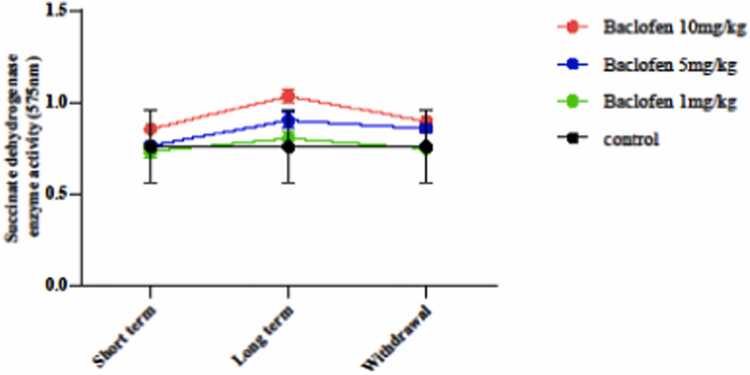
Effects of GABA receptor agonist (1, 5, and 10 mg/kg) in short-term administration (1 day), long-term administration (7 days), and withdrawal syndrome (eight days) on the activity of mitochondrial succinate dehydrogenase by MTT assay in the mouse brain. Values are mean ± SD (n = 6 mice).

Succinate dehydrogenase activity was measured using an MTT assay. As shown in Fig. 8, succinate dehydrogenase activity in all groups receiving baclofen at different exposure periods did not differ significantly from that in the control group.

#### 3.2.5 Effect of baclofen on protein carbonyl level

**Fig. 9:**
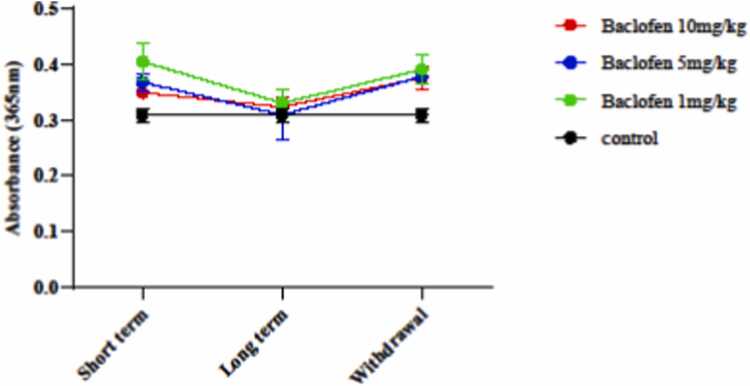
Effects of GABA receptor agonist (1, 5, and 10 mg/kg) in short-term administration (1 day), long-term administration (7 days), and with the drawal syndrome (eight days) on protein carbonyl (PC) in the mouse brain. Values are mean ± SD (n = 6 mice).

DNPH calculated **protein carbonyl** content. As shown in Fig. 9, protein carbonyl levels in all baclofen-treated groups across different exposure periods did not differ significantly from those in the control group.

## 4 Discussion

This study investigated the effect of GABAergic receptor (GABA_B_) stimulation by baclofen on the pain threshold, seizure threshold, and oxidative damage in the brain mitochondria of mice. The results showed that baclofen at the highest dose (10 mg/kg) significantly increased pain tolerance threshold on the first and seventh days after injection. Tail-flick and hot-plate tests both showed that baclofen at 10 mg/kg on the seventh day of administration could increase the duration of response compared to the short-term (1 day) and withdrawal syndrome (eighth day) (Fig. 1, Fig. 2). Previous studies demonstrated that the use of baclofen can increase the duration of response to a painful stimulus using the tail flick test [41].

Evaluation of locomotor activity by the rotarod experiment (Fig. 4) showed that baclofen at 10 mg/kg on the first day of injection and at 1 and 5 mg/kg on the seventh day of injection caused a significant decrease in balance, power, and motor coordination in mice. Only a high dose of baclofen (10 mg/kg) could reduce locomotor activity in mice in the short term (1 day). When the treatment period is extended to the seventh day, lower doses (1 and 5 mg/kg) also affect balance and motor power in the animals. It can be concluded that the effect of baclofen on motor performance using the rotarod apparatus is time and dose-dependent. Therefore, baclofen can decrease the balance and motor power, which in turn causes a delay in the response to the pain stimulus, so it’s not a painkiller drug [39]. Cryan JF et al. studied motor power in animals treated with baclofen using the rotarod test and concluded that baclofen decreased motor balance and motor power [11]. Pain is driven by glutamate, the primary excitatory neurotransmitter, in the nervous system. GABA, the primary inhibitory neurotransmitter, can counteract glutamate's effects, thereby alleviating pain. Still, it can also excessively inhibit brain areas responsible for motor coordination, which may account for the results observed in the rotarod test [34].

Moreover, baclofen is a known muscle relaxant; the prolonged latency to pain stimuli may reflect motor impairment rather than an actual analgesic effect.

It was stated that the Imperfections in the GABA breakdown can frequently induce seizures. Baclofen increased the seizure threshold in a dose- and time-dependent manner (Fig. 3) compared with the control group. The highest dose of baclofen (10 mg/kg) on the first day of administration significantly increased the seizure threshold. All doses (1, 5, and 10 mg/kg) during the seventh day of the injection period (long-term exposure) increased the seizure threshold compared to the control group. Baclofen (10 mg/kg) could significantly increase the seizure threshold on the eight days (the withdrawal syndrome) compared to the control group. The results showed that the antiseizure effects of baclofen depend on the amount of drug consumed and the duration of exposure. In addition, a human study found that baclofen use is associated with seizure-inducing effects, which differ from the results of the present study. Baclofen's potential to trigger seizures is believed to vary with age, showing a higher risk in younger individuals, although this aspect remains debated. Alterations in molecular or neuronal network functions that affect GABAergic signaling via receptor activity may increase neuronal excitability, a hallmark of epileptogenic tissue. Factors such as changes in intrinsic membrane properties, shifts in the balance between excitation and inhibition within neuronal networks, or enhanced synchronization of neuronal populations have all been associated with the development of epilepsy [16]. It was stated that the use of 1 and 5 mg/kg of baclofen in different age groups of rats demonstrated that baclofen can control seizures induced by pentylenetetrazole in a dose- and age-dependent manner [43].

In this study, baclofen had protective effects against oxidative damage induced by oxidant agents, primarily under long-term exposure (7 days) (Fig. 5, Fig. 6, Fig. 7, Fig. 8, Fig. 9). The effects of baclofen on ROS production depend on the drug dose and the duration of exposure (Deng, Li et al.,. In this study, the highest dose (10 mg/kg) and long-term exposure to baclofen were found to affect ROS levels. Still, neither low nor medium doses in long-term exposure, nor any dose in short-term exposure, nor withdrawal syndrome (eight days) affects ROS levels. Fig. 3 demonstrates that the seizure threshold stays elevated up to the 8th day after withdrawal, whereas Fig. 5, Fig. 6 reveal that ROS and MDA levels have returned to baseline. This dissociation between functional and biochemical measures is intriguing and suggests the involvement of mechanisms beyond oxidative stress. Behavioral flexibility is reduced following interventions that interfere with GABAergic signaling, such as neonatal ventral hippocampal lesions, GABAA receptor blockade in the medial prefrontal cortex (mPFC), and genetic impairments of GABAergic interneurons. Acute systemic administration of baclofen enhances behavioral flexibility, suggesting that this drug could help treat clinical conditions characterized by impaired behavioral flexibility [5]. Neuroplasticity refers to the nervous system's capacity to adapt to internal or external stimuli by reshaping its structure, function, and connections. Baclofen administration increases neuroplasticity and improves functional recovery in a stroke model [18]. Activation of GABAergic signaling through the GABA_B_ receptor agonist baclofen can enhance behavioral flexibility, and GABA_B_ receptor agonists could be beneficial for managing behavioral impairments in neuropsychiatric conditions. The capacity to adjust one’s actions flexibly in reaction to environmental changes is a vital component of normal adaptive behavior, supported by the prefrontal cortex [4], [18].

This study supports other studies that showed baclofen reduces oxidative stress. The GABAergic pathway plays an essential role in the improvement of many diseases through lowering oxidative damage and ROS by the neuronal and intracellular signal transduction pathway [45]. The GABA_B_ receptor reduces damage in the gastrointestinal tract via intracellular and neuroendocrine signaling pathways. Baclofen activates GABA_B_ receptors, which leads to the release of neurotransmitters and reduces the production of ROS, and finally provides a protective effect [15]. The results of Li Liu et al. were in agreement with the present study, which showed chronic administration of baclofen significantly reduced nerve tissue damage by reducing oxidative stress biomarkers, regulating Bcl-2/Bax ratio, increasing the activation of Akt, GSK-3β, and ERK, which led to the suppression of cellular destructive autophagy [24].

More severe baclofen toxicity can manifest as generalized tonic–clonic or myoclonic seizures, despite its CNS inhibitory effects. These seizures may arise from its influence on both GABAergic and glutamatergic pathways. Moreover, increased GABA_B_ receptor activity can lead to neural excitation by hyperpolarizing presynaptic and postsynaptic inhibitory interneurons, thereby lowering the seizure threshold [33]. Baclofen significantly decreases the release of IL-6 and IL-12 in lipopolysaccharide (LPS)-induced inflammation in mouse microglial cells. Additionally, baclofen reduces IL-6 and TNF-α secretion, as well as NF-κB and p38 MAPK activation, in both astrocytes and microglia during inflammation triggered by lipopolysaccharide and interferon-γ [19]. In the pentylenetetrazol-induced epilepsy model, acylated ghrelin demonstrated a neuroprotective effect by inhibiting the JNK-mediated mitochondrial apoptosis pathway. Furthermore, the combined use of baclofen and muscimol—respectively targeting ionic and metabolic GABA receptors-attenuated JNK-driven neuronal apoptosis by disrupting the Glu6R-PSD95-MLK3 signaling complex [19]. GABA significantly inhibits the TLR4/MyD88/NLRP3 pathway by activating the GABA_B_ receptor, which, in turn, enhances NRF2/HO-1 signaling. This process increases levels of antioxidant molecules and enhances antioxidant capacity in both mice and IPEC-J2 cells [13].

A high dose of baclofen (10 mg/kg) administered in long-term exposure resulted in a significant decrease in lipid peroxidation in the animals. All doses (1, 5, and 10 mg/kg) on long-term exposure, and the low and medium doses during the long-term period, are ineffective in reducing lipid peroxidation. The withdrawal syndrome (eight days) in all doses showed no effect on the level of MDA. Previous studies have investigated the effect of baclofen on lipid oxidation, reporting that it can reduce MDA formation [35].

Baclofen at a high dose (10 mg/kg) and long-term exposure significantly increase GSH levels compared to the control group. The increase in GSH content by baclofen is dose and time-dependent. Morley KC et al. showed that baclofen reduced GSH levels and stated that baclofen at 10 mg/kg led to a significant increase in GSH content [28].

Given that in this study all groups were anesthetized with ketamine/xylazine (10/1) (per the ethical protocol) and then sacrificed, this bias was present across all groups (including the control group), and this was one of the limitations of our study. The use of ketamine before animal sacrifice can cause a strong block of glutamate receptors (ligand-gated ion channels called NMDA receptors) and may affect oxidative stress biomarkers, as oxidative stress can result from excitotoxicity induced by high glutamate levels. Ketamine can decrease neuronal excitability and may also alleviate pain by affecting other pathways [1]. Co-administration of ketamine and baclofen requires caution and close medical supervision to monitor for excessive sedation and respiratory issues. The combination is likely to increase CNS depression and could alter the pharmacodynamic responses of baclofen due to ketamine's influence on GABA_B_ receptor signaling [2]. Choosing the correct dose of baclofen in the study is very important. Baclofen toxicity or overdose causes seizures, including generalized tonic-clonic, myoclonus, and status epilepticus [14]. In clinical studies, loss of seizure control has been reported in patients receiving baclofen; patients with a seizure history should be monitored carefully. Baclofen has central nervous system depressant effects, causing sedation, dizziness, and weakness, which can confound the interpretation of neurological symptoms in seizure studies [48].

Baclofen, a selective GABA_B_ receptor agonist, has been shown to decrease oxidative stress through enhancing antioxidant defenses and reducing inflammatory pathways, such as the TLR4/MyD88/NLRP3 pathway. These antioxidant and anti-inflammatory properties are linked to neuroprotection and improved cell viability in various diseases.

## 5 Conclusion

Baclofen, a GABAB receptor agonist, increases the seizure threshold in a dose- and time-dependent manner. It was also found that baclofen reduces oxidative damage by decreasing ROS and MDA levels and increasing glutathione content. Baclofen's effects on oxidative stress and seizure modulation offer promising insights for future therapeutic strategies in epilepsy, chronic pain, and other neurological diseases. By the way, baclofen: understanding the exact mechanism(s) involved in the seizures and mitochondrial dysfunction it causes requires more extensive studies.

## CRediT authorship contribution statement

**Mohammad Seyedabadi:** Visualization, Formal analysis, Conceptualization. **Fatemeh Nasiri:** Methodology, Investigation. **Roghayeh Jahani:** Writing – original draft, Methodology. **Paria Pourbahram:** Methodology, Investigation. **Hamidreza Mohammadi:** Writing – review & editing, Funding acquisition, Formal analysis, Data curation, Conceptualization.

## Ethics approval statement

Ethics approval for this study was obtained from Mazandaran University of Medical Sciences, Sari, Iran. Approval ID: IR. MAZUMS.REC.1400.8929

### Funding

Financial support for this work was provided by Mazandaran University of Medical Sciences, Sari, Iran, with Approval ID: IR. MAZUMS.REC.1400.8929–18127

## Declaration of Competing Interest

The authors declare that they have no known competing financial interests or personal relationships that could have appeared to influence the work reported in this paper.

## Data Availability

All data are available and accessible in the manuscript text.

## References

[1] Aleksandrova L.R., Phillips A.G., Wang Y.T. (2017). Antidepressant effects of ketamine and the roles of AMPA glutamate receptors and other mechanisms beyond NMDA receptor antagonism. J. Psychiatry Neurosci..

[2] Ando Y., Hojo M., Kanaide M., Takada M., Sudo Y., Shiraishi S., Sumikawa K., Uezono Y. (2011). S (+)-ketamine suppresses desensitization of γ-aminobutyric acid type B receptor-mediated signaling by inhibition of the interaction of γ-aminobutyric acid type B receptors with G protein-coupled receptor kinase 4 or 5. Anesthesiology.

[3] Anwar H., Khan Q.U., Nadeem N., Pervaiz I., Ali M., Cheema F.F. (2020). Epileptic seizures. Discoveries.

[4] Beas B., McQuail J., Bañuelos C., Setlow B., Bizon J. (2017). Prefrontal cortical GABAergic signaling and impaired behavioral flexibility in aged F344 rats.". Neuroscience.

[5] Beas B.S., Setlow B., Bizon J.L. (2016). Effects of acute administration of the GABA (B) receptor agonist baclofen on behavioral flexibility in rats. Psychopharmacology.

[6] Benson M.J., Anderson L.L., Low I.K., Luo J.L., Kevin R.C., Zhou C., McGregor I.S., Arnold J.C. (2022). Evaluation of the possible anticonvulsant effect of Δ9-tetrahydrocannabinolic acid in murine seizure models. Cannabis Cannabinoid Res..

[7] Bradford M.M. (1976). A rapid and sensitive method for the quantitation of microgram quantities of protein utilizing the principle of protein-dye binding. Anal. Biochem..

[8] Brandenburg J.E. (2023). Is baclofen the least worst option for spasticity management in children?". J. Pediatr. Rehabil. Med..

[9] Carlson S.L., O’Buckley T.K., Thomas R., Thiele T.E., Morrow A.L. (2014). Altered GABA A receptor expression and seizure threshold following acute ethanol challenge in mice lacking the RIIβ subunit of PKA.". Neurochem. Res..

[10] Chartier M., Malissin I., Tannous S., Labat L., Risède P., Mégarbane B., Chevillard L. (2018). Baclofen-induced encephalopathy in overdose–modeling of the electroencephalographic effect/concentration relationships and contribution of tolerance in the rat.". Prog. NeuroPsychopharmacol. Biol. Psychiatry.

[11] Cryan J.F., Kelly P.H., Chaperon F., Gentsch C., Mombereau C., Lingenhoehl K., Froestl W., Bettler B., Kaupmann K., Spooren W.P. (2004). Behavioral characterization of the novel GABAB receptor-positive modulator GS39783 (N, N′-dicyclopentyl-2-methylsulfanyl-5-nitro-pyrimidine-4, 6-diamine): anxiolytic-like activity without side effects associated with baclofen or benzodiazepines. J. Pharmacol. Exp. Ther..

[12] De Beaurepaire R., Sinclair J.M., Heydtmann M., Addolorato G., Aubin H.-J., Beraha E.M., Caputo F., Chick J.D., de La Selle P., Franchitto N. (2019). The use of baclofen as a treatment for alcohol use disorder: a clinical practice perspective. Front. Psychiatry.

[13] Deng Z., Li D., Wang L., Lan J., Wang J., Ma Y. (2024). Activation of GABABR attenuates intestinal inflammation by reducing oxidative stress through modulating the TLR4/MyD88/NLRP3 pathway and gut microbiota abundance. Antioxidants.

[14] Dietz N., Wagers S., Harkema S.J., D'Amico J.M. (2023). Intrathecal and oral baclofen use in adults with spinal cord injury: a systematic review of efficacy in spasticity reduction, functional changes, dosing, and adverse events. Arch. Phys. Med. Rehabil..

[15] Gao L., Zhao H., Zhu T., Liu Y., Hu L., Liu Z., Huang H., Chen F., Deng Z., Chu D. (2017). The regulatory effects of lateral hypothalamus area GABAB receptor on gastric ischemia-reperfusion injury in rats. Front. Physiol..

[16] Hansel D.E., Hansel C.R., Shindle M.K., Reinhardt E.M., Madden L., Levey E.B., Johnston M.V., Hoon Jr A.H. (2003). Oral baclofen in cerebral palsy: possible seizure potentiation?". Pediatr. Neurol..

[17] Hinarejos I., Machuca C., Sancho P., Espinós C. (2020). Mitochondrial dysfunction, oxidative stress and neuroinflammation in neurodegeneration with brain iron accumulation (NBIA).". Antioxidants.

[18] Hodor A., Palchykova S., Baracchi F., Noain D., Bassetti C.L. (2014). Baclofen facilitates sleep, neuroplasticity, and recovery after stroke in rats. Ann. Clin. Transl. Neurol..

[19] Hyder Pottoo F., Salahuddin M., Khan F.A., Albaqshi B.T., Gomaa M.S., Abdulla F.S., AlHajri N., Alomary M.N. (2022). Trio-drug combination of sodium valproate, baclofen and thymoquinone exhibits synergistic anticonvulsant effects in rats and neuro-protective effects in HEK-293 cells. Curr. Issues Mol. Biol..

[20] Inaltekin A., Kivrak Y. (2021). Evaluation of the effect of vortioxetine on pain threshold by hot-plate test in mice. Arch. Neuropsychiatry.

[21] Jirsa V.K., Stacey W.C., Quilichini P.P., Ivanov A.I., Bernard C. (2014). On the nature of seizure dynamics. Brain.

[22] Keyhanfar F., Meymandi M.S., Sepehri G., Rastegaryanzadeh R., Heravi G. (2013). Evaluation of antinociceptive effect of pregabalin in mice and its combination with tramadol using tail flick test. Iran. J. Pharm. Res. IJPR.

[23] Kumar P., Liu C., Hsu J.W., Chacko S., Minard C., Jahoor F., Sekhar R.V. (2021). Glycine and N-acetylcysteine (GlyNAC) supplementation in older adults improves glutathione deficiency, oxidative stress, mitochondrial dysfunction, inflammation, insulin resistance, endothelial dysfunction, genotoxicity, muscle strength, and cognition: results of a pilot clinical trial. Clin. Transl. Med..

[24] Liu L., Li C.-j, Lu Y., Zong X.-g, Luo C., Sun J., Guo L.-j (2015). Baclofen mediates neuroprotection on hippocampal CA1 pyramidal cells through the regulation of autophagy under chronic cerebral hypoperfusion.". Sci. Rep..

[25] Mannix R., Berglass J., Berkner J., Moleus P., Qiu J., Andrews N., Gunner G., Berglass L., Jantzie L.L., Robinson S. (2014). Chronic gliosis and behavioral deficits in mice following repetitive mild traumatic brain injury. J. Neurosurg..

[26] Mohammadnejad L., Soltaninejad K., Seyedabadi M., Ghasem Pouri S.K., Shokrzadeh M., Mohammadi H. (2021). Evaluation of mitochondrial dysfunction due to oxidative stress in therapeutic, toxic and lethal concentrations of tramadol. Toxicol. Res..

[27] Momtaz S., Lall N., Hussein A., Ostad S.N., Abdollahi M. (2010). Investigation of the possible biological activities of a poisonous South African plant; Hyaenanche globosa (Euphorbiaceae). Pharmacogn. Mag..

[28] Morley K.C., Lagopoulos J., Logge W., Chitty K., Baillie A., Haber P.S. (2018). Neurometabolite levels in alcohol use disorder patients during baclofen treatment and prediction of relapse to heavy drinking. Front. Psychiatry.

[29] Nazaré M.S.L. d, Silva J.A.M.G., Navega M.T., Fagnello-Navega F.R. (2014). Comparison of pain threshold and duration of pain perception in men and women of different ages. Fisioter. em Mov..

[30] Otero-Luis I., Saz-Lara A., Cavero-Redondo I., Pascual-Morena C., Martínez-García I., de Arenas-Arroyo S.N. (2025). Effectiveness of the intrathecal baclofen pump in the treatment of spasticity of different aetiologies: A systematic review and meta-analysis. Neurol. ía.

[31] Perveen N., Alqahtani F., Ashraf W., Rasool M.F., Anjum S.M.M., Kaukab I., Ahmad T., Alqarni S.A., Imran I. (2024). Perampanel increases seizure threshold in pentylenetetrazole-kindled mice and improves behavioral dysfunctions by modifying mRNA expression levels of BDNF/TrkB and inflammatory markers. Saudi Pharm. J..

[32] Redondo-Orúe I., Sánchez-Baena S., Paret-Fernández A., Rodríguez-Costa I., Morales C.R., López-López D., Pecos-Martín D., de la Flor Á.G. (2025). Differences on lower trapezius pressure pain threshold, muscle strength and muscle thickness in individuals with chronic neck pain and active or latent myofascial triggers points. J. Tissue Viability.

[33] Romito J.W., Turner E.R., Rosener J.A., Coldiron L., Udipi A., Nohrn L., Tausiani J., Romito B.T. (2021). Baclofen therapeutics, toxicity, and withdrawal: a narrative review. SAGE Open Med..

[34] Saffarpour S., Shaabani M., Naghdi N., Farahmandfar M., Janzadeh A., Nasirinezhad F. (2017). In vivo evaluation of the hippocampal glutamate, GABA and the BDNF levels associated with spatial memory performance in a rodent model of neuropathic pain. Physiol. Behav..

[35] Samotrueva M., Magomedov M., Khlebtsova E., Tiurenkov I. (2011). Influence of gaba derivatives on some indices of lipid peroxidation in immunocompetent organs under experimental immunopathology conditions. Eksperimental’naia i Klinicheskaia Farmakologiia.

[36] Sarlo G.L., Holton K.F. (2021). Brain concentrations of glutamate and GABA in human epilepsy: a review. Seizure.

[37] Schmidt R., Welzel B., Merten A., Naundorf H., Löscher W. (2025). Temporal development of seizure threshold and spontaneous seizures after neonatal asphyxia and the effect of prophylactic treatment with midazolam in rats.". Exp. Neurol..

[38] Schmitz N.S., Krach L.E., Coles L.D., Mishra U., Agarwal S.K., Cloyd J.C., Kriel R.L. (2017). A randomized dose escalation study of intravenous baclofen in healthy volunteers: clinical tolerance and pharmacokinetics. PMR.

[39] Serrano-Regal M.P., Bayón-Cordero L., Chara Ventura J.C., Ochoa-Bueno B.I., Tepavcevic V., Matute C., Sánchez-Gómez M.V. (2022). GABAB receptor agonist baclofen promotes central nervous system remyelination. Glia.

[40] Slade G.D., Sanders A.E., Ohrbach R., Fillingim R.B., Dubner R., Gracely R.H., Bair E., Maixner W., Greenspan J.D. (2014). Pressure pain thresholds fluctuate with, but do not usefully predict, the clinical course of painful temporomandibular disorder. PAIN.

[41] Smith D. (1984). Stereoselectivity of spinal neurotransmission: Effects of baclofen enantiomers on tail-flick reflex in rats. J. Neural Transm..

[42] van Dijk L.M., van Zwol A., Buizer A.I., van de Pol L.A., Slot K.M., de Wildt S.N., Bonouvrié L.A. (2024). Potentially Life-Threatening Interaction between Opioids and Intrathecal Baclofen in Individuals with a Childhood-Onset Neurological Disorder: A Case Series and Review of the Literature. Neuropediatrics.

[43] Velíšková J., Velíšek L., Moshé S.L. (1996). Age-Specific effects of baclofen on Pentylenetetrazol-Induced seizures in developing rats. Epilepsia.

[44] Xie M., Qin H., Liu L., Wu J., Zhao Z., Zhao Y., Fang Y., Yu X., Su C. (2025). GABA regulates metabolic reprogramming to mediate the development of brain metastasis in non-small cell lung cancer. J. Exp. Clin. Cancer Res..

[45] Xu B., Feng X., Piechatzek A., Zhang S., Konrad K.R., Kromdijk J., Hedrich R., Gilliham M. (2024). The GABA shunt contributes to ROS homeostasis in guard cells of Arabidopsis. N. Phytol..

[46] Yang P., Sheng D., Guo Q., Wang P., Xu S., Qian K., Li Y., Cheng Y., Wang L., Lu W. (2020). Neuronal mitochondria-targeted micelles relieving oxidative stress for delayed progression of Alzheimer's disease. Biomaterials.

[47] Yowtak J., Lee K.Y., Kim H.Y., Wang J., Kim H.K., Chung K., Chung J.M. (2011). Reactive oxygen species contribute to neuropathic pain by reducing spinal GABA release. Pain.

[48] Zhang N., Jiang T., Li Y., Guo P., Liu Y., Zhang Y., Liu Y. (2025). Neurological adverse events associated with baclofen: A disproportionality analysis based on FDA Adverse Event Reporting System. SAGE Open Med..

